# Analysis of metabolomic profile of fermented *Orostachys japonicus* A. Berger by capillary electrophoresis time of flight mass spectrometry

**DOI:** 10.1371/journal.pone.0181280

**Published:** 2017-07-13

**Authors:** Gitishree Das, Jayanta Kumar Patra, Sun-Young Lee, Changgeon Kim, Jae Gyu Park, Kwang-Hyun Baek

**Affiliations:** 1 Research Institute of Biotechnology & Medical Converged Science, Dongguk University-Seoul, Ilsandong-gu, Gyeonggi-do, Republic of Korea; 2 Department of Food Science and Technology, Chung-Ang University, Anseong, Gyeonggi-do, Republic of Korea; 3 Pohang Center for Evaluation Biomaterials (POCEB), Pohang Technopark Foundation, Pohang, Gyeongbuk, Republic of Korea; 4 Department of Biotechnology, Yeungnam University, Gyeongsan, Gyeongbuk, Republic of Korea; MJP Rohilkhand University, INDIA

## Abstract

Microbial cell performance in food biotechnological processes has become an important concern for improving human health worldwide. *Lactobacillus plantarum*, which is widely distributed in nature, is a lactic acid bacterium with many industrial applications for fermented foods or functional foods (e.g., probiotics). In the present study, using capillary electrophoresis time of flight mass spectrometry, the metabolomic profile of dried *Orostachys japonicus* A. Berger, a perennial medicinal herb with *L*. *plantarum* was compared with that of *O*. *japonicus* fermented with *L*. *plantarum* to elucidate the metabolomic changes induced by the fermentation process. The levels of several metabolites were changed by the fermentation process, indicating their involvement in microbial performance. For example, glycolysis, the pentose phosphate pathway, the TCA cycle, the urea cycle-related metabolism, nucleotide metabolism, and lipid and amino acid metabolism were altered significantly by the fermentation process. Although the fermented metabolites were not tested using *in vivo* studies to increase human health benefits, our findings provide an insight into the alteration of metabolites induced by fermentation, and indicated that the metabolomic analysis for the process should be accompanied by fermenting strains and conditions.

## Introduction

Lactic acid bacteria are gram-positive bacteria that generate lactic acid as a main end product of carbohydrate metabolism; therefore, they are used heavily in industry and homes for dairy fermentation. A record of safe use of lactobacilli is broadly accepted; and currently, specific *Lactobacillus* strains associated with health benefits are sold as probiotics [1]. *Lactobacilli*, which comprise about 90 species and subspecies, ferment various carbohydrates, such as glucose, sucrose, fructose, and galactose, mainly to lactate and acetate. *Lactobacillus* sp., as probiotics, are used as organic control agents for human health and aquaculture. They produce bacteriocin, which inhibits the growth of gram-positive and gram-negative bacteria, including *Escherichia coli*, *Staphylococcus aureus*, *Pseudomonas aeruginosa*, and *Listeria innocua* [2].

*Lactobacillus* species is distributed widely in nature [3] and is a facultative hetero-fermentative lactic acid bacterium [4]. This bacterium is a part of the adventitious or first course microbiota of fruit, starchy, dairy, cereal foods, meat, vegetables, and fish products [5–7]. *L*. *plantarum* completes the ultimate phase of vegetable and natural fruit fermentations because of its elevated acid tolerance compared with other lactic acid microorganisms [2, 8]. *L*. *plantarum* strains are present in the human gastrointestinal tract and are important for their immunomodulatory effects and therapeutic applications [9, 10].

Metabolites are the final products of cellular regulatory progression, and their levels can be regarded as the ultimate response of biological systems to genetic or ecological alterations. Metabolomics aims to discover all the metabolites produced by a living organism [11, 12]. Metabolomics is a recent omics technology that enables the scrutiny of cellular functions through a holistic view of metabolic pathways. Through evaluating the metabolites in samples from an individual, it is possible to gain an insight into the genetic, ecological, and developmental modulators that differentiate the samples. Among diverse metabolite measurement techniques, capillary electrophoresis time of flight mass spectrometry (CE-TOF-MS) is suitable for the concurrent profiling of energy metabolic pathways [13]. Metabolite profiling in fermented foods is used to monitor metabolite alterations throughout fermentation and to determine the nutritional quality of the final fermented product [14]. Metabolomic analyses has importance in respect to the comparative alteration in metabolite profusion in case of relative researches [15]. *Orostachys japonicus* A. Berger is a medicinal herb with immunoregulatory, anti-cancer, and many other therapeutic activities [16]. It is a member of the family Crassulaceae. Extracts of *O*. *japonicus* have been used as a folk remedy for cancer management and also as an adjuvant or an alternative treatment to chemotherapy [17, 18]. Its anti-cancer properties might be derived from its abundant flavonoids [18].

From the early human civilization, microbial metabolism was exploited to produce fermented foods and beverages [19]. Well-known metabolically derived products from microbes include curd, meats, bread, beer, and cheese. [20]. Fermentation of herbal plants with lactic acid bacteria increases various bioactive properties and the levels of beneficial metabolites [21]. Daily intake of probiotic *L*. *plantarum* strains in functional foods are reported to reduce the effect of intestinal disorders [22]. The increasing industrial application of *L*. *plantarum* requires an understanding of its metabolomic profile for human health [23].

In the present study, the metabolic changes in *O*. *japonicus* fermented by *L*. *plantarum* were investigated extensively using capillary electrophoresis time of flight mass spectrometry (CE-TOF-MS). To date, there has been no reported effort to increase the anti-cancer properties of *O*. *japonicus* using fermentation. This is the first report of the massive metabolite profiling of an herb fermented by *L*. *plantarum*.

## Materials and methods

### Microorganisms, plant materials, and culture conditions

The bacterial strain *L*. *plantarum* ATCC 14917T was obtained from the American Type Culture Collection (ATCC, Manassas, Virginia, USA). The plant sample *O*. *japonicus* A Berger was collected from the garden of the World Farmland (Daegu, Republic of Korea), frozen in a deep freezer (MDF-U53V, Sanyo Electric Co., Ltd., Moriguchi, Osaka prefecture, Japan) and freeze-dried for 72 h using a freeze-dryer (FreeZone 6 Litre Console Freeze Dry System, Labconco Corp, Kansas City, KS, USA).

To extract metabolites, *L*. *plantarum* was grown in de Man, Rogosa, and Sharpe (MRS) medium (Becton, Dickinson and Company Sparks, MD, USA) at 28°C at 160 rpm for 72 h. The dried *O*. *japonicus* A Berger was powdered and divided into six equal parts (100 mg each) in 50 mL Falcon tubes. The treatments comprised *O*. *japonicas-*powdered samples that were added 20 mL of MRS media in a tube followed by adding 100 μL of freshly grown *L*. *plantarum* culture containing 1 × 10^7^ colony forming unit per mL of the bacteria and incubated at 28°C with continuous shaking at 160 rpm for 4 days. The controls were *O*. *japonicas-*powders mixed with the fully-grown *L*. *plantarum* in MRS media under the same incubation condition.

### Preparation of samples for metabolic profiling

After 4 days of fermentation, both the controls and the treatments were kept on ice for 5–10 min and centrifuged at 3,000 rpm at 4°C for 20 min. The supernatants were transferred to new Falcon tubes, mixed with an equal volume of ethyl acetate, and shaken for 2 h at room temperature. The pellets were retained for further processing. The supernatants and ethyl acetate was separated using a separating funnel, and then the ethyl acetate layer was acquired and dried using a rotary evaporator (EYELA, Shanghai Eyela Co. Ltd., China). High performance liquid chromatography (HPLC) grade methanol (1.6 mL) was added to dissolve the dried compounds, which were stored on ice.

To the pellets, 10 mL of Milli-Q water was added, and they were washed by vortexing for 30 sec. The samples were centrifuged at 3000 rpm for 5 min and the supernatant was discarded. A methanol extract solution (1.6 mL) was added to both treatment and control tubes, respectively. In the control tubes, the *O*. *japonicus* A Berger samples were added to the control tubes just before extraction of the metabolites. The pellets in the Falcon tubes dissolved by sonication for 30 sec, and 1.1 mL of internal standard solution was added to each tube and mixed well. After mixing, the tubes were left at room temperature for 30 sec and centrifuged at 3000 rpm for 20 min at 4°C. The supernatant was transferred to a 5-kDa-cutoff ultra-centrifugal filter unit (Ultrafree-MC-PLHCC-HMT; Human Metabolome Technologies Inc, Japan) and centrifuged at 9,000 rpm at 4°C for 2–5 h until no liquid remained in the filter cup. The solution was then dried under vacuum conditions at 1,500 rpm for 3 h. After complete drying of the filtered samples, the samples were stored at −80°C until CE-TOF-MS metabolite analysis.

### CE-TOF-MS analysis

The samples were subjected to CE-TOF-MS analysis and the compounds were measured in the cation and anion mode of metabolome analysis. To improve the CE-TOF-MS analysis quality, the samples were diluted 20-fold for measurement. The analysis was carried out according to the procedure described earlier [24, 25, 26]. The cationic metabolites study was carried out with the help of an Agilent CE-TOF-MS system (Agilent Technologies, Santa Clara, CA, USA) with a fused silica capillary of 50 μm × 80 cm. The investigative conditions were, run buffer: cation buffer solution (p/n: H3301-1001); rinse buffer: cation buffer solution (p/n: H3301-1001); sample injection: pressure injection 50 mbar, 10 sec; CE voltage: positive, 27 kV; MS ionization: ESI positive; MS capillary voltage: 4,000 V; MS scan range: m/z 50–1,000; sheath liquid: HMT sheath liquid (p/n: H3301-1020).

The anionic metabolites study was carried out with the same Agilent CE-TOF-MS system with a fused silica capillary of. 50 μm × 80 cm. The investigative conditions were as follows: run buffer: anion buffer solution (p/n: I3302-1023); rinse buffer: anion buffer solution (p/n: I3302-1023); sample injection: pressure injection 50 mbar, 25 sec; CE voltage: positive, 30 kV; MS ionization: ESI Negative; MS capillary voltage: 3,500 V; MS scan range: m/z 50–1,000; sheath liquid: HMT sheath liquid (p/n: H3301-1020).

### Analysis of the metabolomic data

Peaks detected in CE-TOF-MS analysis were extracted using the automatic integration software (Master Hands ver. 2.16.0.15, Keio University; Tokyo, Japan). To obtain peak information, including *m/z*, migration time (MT), and peak area, the peak area was converted to the relative peak area. Putative metabolites were then allocated from the Human Metabolome Technologies (HMT) metabolite database on the basis of m/z and MT. The tolerance was ±0.5 min in MT and ±10 ppm in m/z. Absolute quantification was carried out for 110 metabolites including glycolysis and TCA cycle intermediates, amino acids, and nucleic acids. All the metabolite concentrations were calculated by normalizing the peak area of each metabolite with respect to the area of the internal standard and using standard curves, which were obtained by single-point (100 μM) calibrations. Hierarchical cluster analysis (HCA) was performed by PeakStat ver. 3.18 and Principal component analysis (PCA) was performed by SampleStat ver. 3.14. Data analyses and visualization were conducted using GraphPad Prism (version 6.0).

## Results and discussion

Metabolism is the most essential process for maintaining life, and refers to those biochemical pathways that either generate biologically functional energy or use that energy to allow growth. Catabolism converts chemical energy into high-energy ATP bonds. Cells produce ATP using two distinct mechanisms: oxidative phosphorylation and substrate level phosphorylation. In substrate level phosphorylation, ATP is synthesized as a result of converting an organic molecule from one form to another [27].

Live lactic acid bacteria consumption and fermented foods have been a habitual part of human food intake for a long time [9]. By the breakdown of indigestible components, detoxification of compounds in the diet, and the production of vitamins, beneficial live bacteria can stimulate appetite and improve nourishment [2]. These beneficial live bacteria have been termed “probiotics”. The concept behind the probiotics is the equilibrium between valuable and harmful microorganisms in the microflora [9]; although, currently, probiotics are used generally to describe live bacteria that maintain health and have valuable effects beyond conventional nutrition; some of these health advantageous properties have been proven scientifically [9].

**Fig 1:**
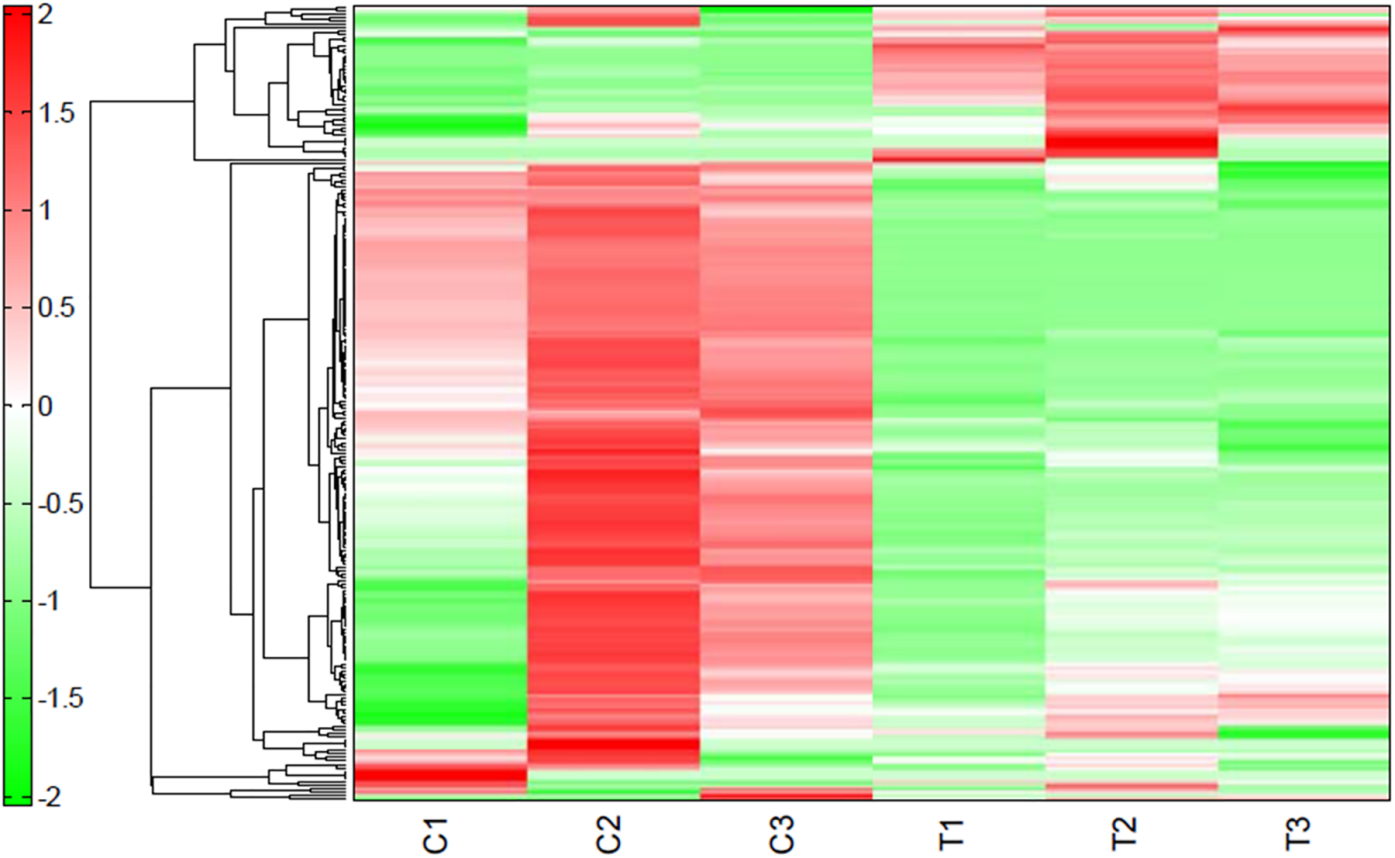
A heat map of hierarchical cluster analysis (HCA) comparing the altered metabolites between of *O*. *japonicus* fermented with *L*. *plantarum* and those of *O*. *japonicus* added with *L*. *plantarum*. The x-axis shows the controls (C1, C2, and C3) for the metabolites in *O*. *japonicus* added with *L*. *plantarum* and the treatments (T1, T2, and T3) for those in *O*. *japonicus* fermented with *L*. *plantarum*. The y-axis indicates the heat map for the change of metabolites. The distances between peaks are displayed in tree diagrams, and the degrees of red color and of green color indicate higher and lower contents of the metabolites, respectively.

The lactic acid bacterium *L*. *plantarum* is an industrially significant microbe used globally, especially in industrial food fermentations [28]. To manufacture quality food products, *L*. *plantarum* plays a vital role in numerous food-processing industries [29]. To determine the promising active metabolites produced by *L*. *plantarum* fermentation on the medicinal herb *O*. *japonicus*, we explored the metabolomic changes using CE-TOF-MS (Fig 1). The metabolite profiling of *L*. *plantarum* just added to *O*. *japonicus* (control) and *L*. *plantarum* grown in presence of *O*. *japonicus* (treatment) was carried out using CE-TOF-MS analysis in two modes for cationic and anionic metabolites.

A total of 230 metabolites (144 and 86 in the cationic and anionic mode, respectively) were detected. Considerable alteration and variation of metabolites was found between *O*. *japonicus* fermented by *L*. *plantarum* compared with metabolites of *O*. *japonicus* with *L*. *plantarum* (Fig 1; S1 Table). The relative peak areas between *L*. *plantarum* fermented *O*. *japonicus* (treatments) and *L*. *plantarum* in presence of *O*. *japonicus* (controls) were calculated for the 230 putative metabolites.

**Fig 2:**
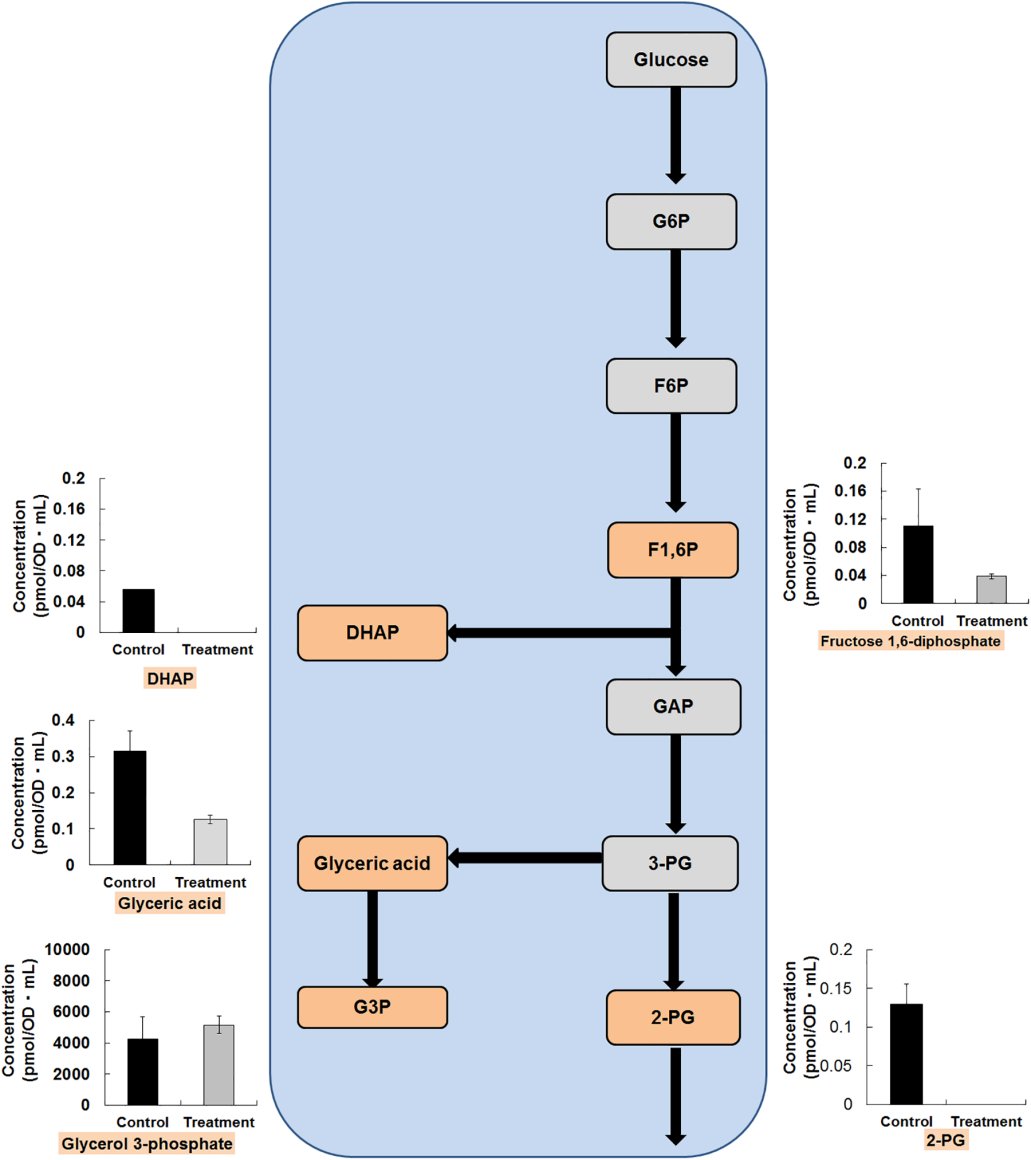
Metabolite changes in the glycolysis pathway by *L*. *plantarum* fermentation. Changes in the metabolite levels of in the fermented *O*. *japonicus* by *L*. *plantarum* (gray bars) compared with those in the *O*. *japonicus* with *L*. *plantarum* (black bars) were detected by capillary electrophoresis time of flight mass spectrometry (CE-TOF-MS). The error bars indicate the mean standard deviation of triplicate samples. The figure also includes a schematic diagram of glycolysis. Abbreviations: G6P, glucose 6-phosphate; F6P, fructose 6-phosphate; F1,6P, fructose 1,6-bisphosphate; G3P, glyceraldehyde 3-phosphate; DHAP, dihydroxyacetone phosphate; 3PG, 3-Phosphoglyceric acid; 2PG, 2-Phosphoglyceric acid.

Glycolytic pathways use glucose as the substrate, since this simple sugar needs the least catalytic steps to go through central metabolism. Although glycolysis can metabolize further carbon sources, acid sugars must first be reduced and sugar alcohols must be oxidized [27]. The changes of metabolites in the glycolytic pathway of *O*. *japonicus* fermented by *L*. *plantarum* are shown in Fig 2. There were considerable reductions in the concentrations of the entire intracellular metabolites of the glycolysis pathway of *O*. *japonicas* fermented by *L*. *plantarum* (Fig 2). For example, the content of fructose 1, 6-diphosphate (F-1, 6-P) was reduced to only 65.19% by fermentation. The contents of dihydroxyacetone phosphate (DHAP) and 2-phosphoglyceric acid (2PG) in the treatments were below the detection limit compared with those of the controls, which was also the case for glucose 6-phosphate (G6P), fructose 6-phosphate (F6P), and 3-phosphoglycerate (3PG). Pyruvic acid (34.95%) and lactic acid (61.56%) were decreased by the *L*. *plantarum* fermentation. In the glycolytic pathway, there was a lower accumulation of intermediates of sucrose and starch. The major purpose of glycolysis is to generate high energy molecules, and pyruvate and intermediate compounds serve as important starters for other cellular process; therefore significant reductions in the metabolites of the glycolysis pathways is evidence of the production of high energy molecules and other intermediate products by the fermentation of *O*. *japonicas*.

**Fig 3:**
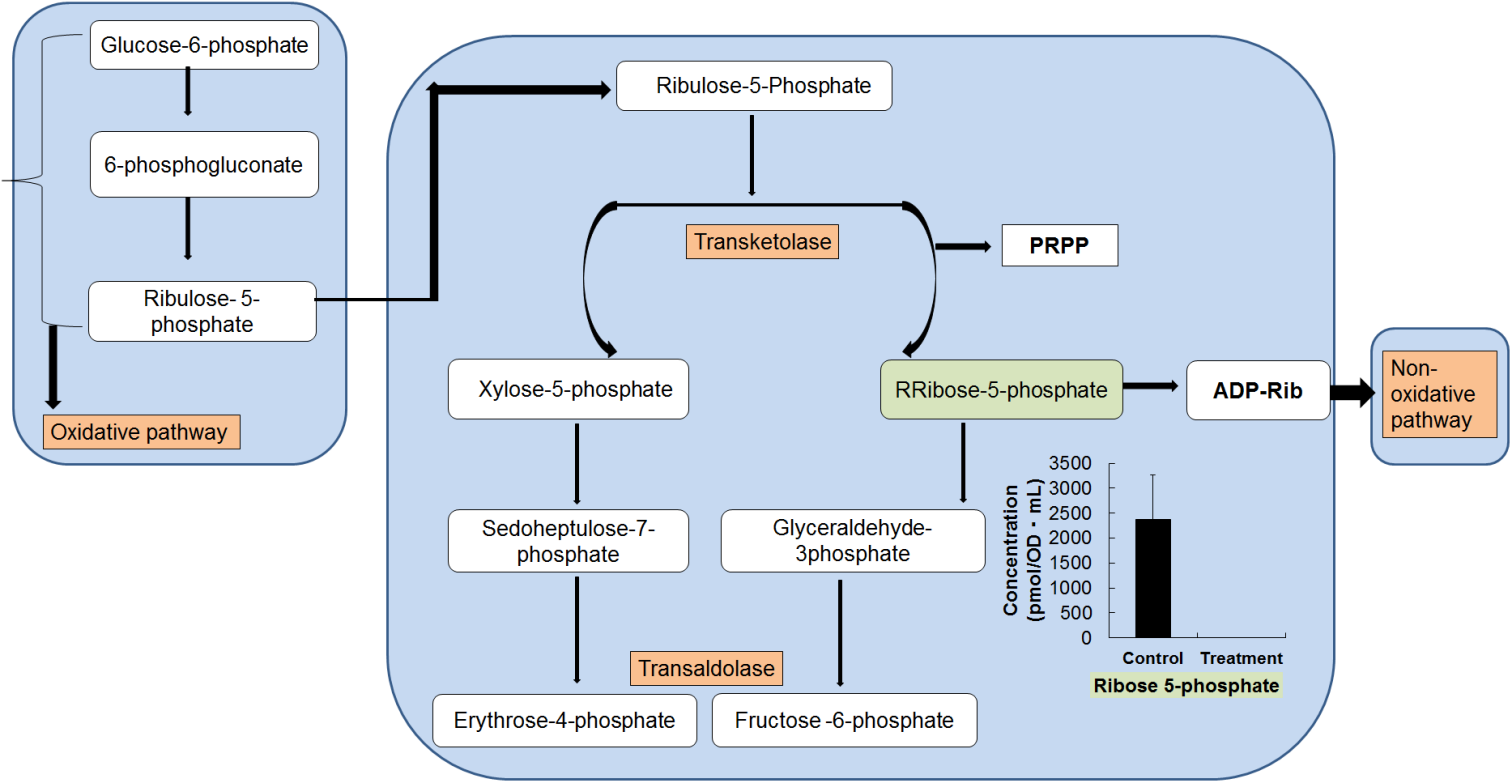
Metabolite changes observed in the pentose phosphate pathway (oxidative and non-oxidative pathway). Changes in the metabolite levels of in the fermented *O*. *japonicus* by *L*. *plantarum* (gray bars) compared with those in the *O*. *japonicus* with *L*. *plantarum* (black bars) acquired by capillary electrophoresis time of flight mass spectrometry (CE-TOF-MS). The error bar indicates the mean standard deviation of triplicate samples. The figure also includes a schematic diagram of the pentose phosphate pathway.

The analysis of intermediate metabolites of the pentose phosphate (PP) pathway is shown in Fig 3. In the PP pathway, oxidized glucose-6-phosphate is converted to pentose phosphates. It is a unique pathway for several reasons. Initially, it uses a diverse set of reactions in the Embden–Meyerhof–Parnas (EMP) pathway and it oxidizes sugars with NADP^+^ rather than NAD^+^. The resultant NADPH is a major source of electrons for various biosynthetic processes. It generates D-ribose-5-phosphate, sedoheptulose-7-phosphate, and erythrose-4-phosphate, which function as precursors for the biosynthesis of amino acids, nucleic acids, and other macromolecules, including ATP, coenzyme A, and NADH. Therefore, the PP pathway is a vital central metabolic pathway. The PP pathway starts with three molecules of one glycolytic intermediate, β-D-glucose 6- phosphate, and ends with formation of three others, two molecules of β-D-fructose-6-phosphate and one of D-glyceraldehyde-3-phosphate [27, 30]. The approach for reducing the pyruvate formed through glycolysis determines the fermentation product and the fermentation pathway name. More significantly, the end product of fermentation determines the balance among the net ATP and net recycled electron acceptors (i.e., NAD^+^ and NADP^+^). In the current study, the maximum metabolites in the PP pathway were under the detection limits in the treated samples. In the PP pathway, lactic acid is the final product [31]. It seems that maximum metabolites of this pathway were completely metabolized, with a higher production of NAD^+^.

**Fig 4:**
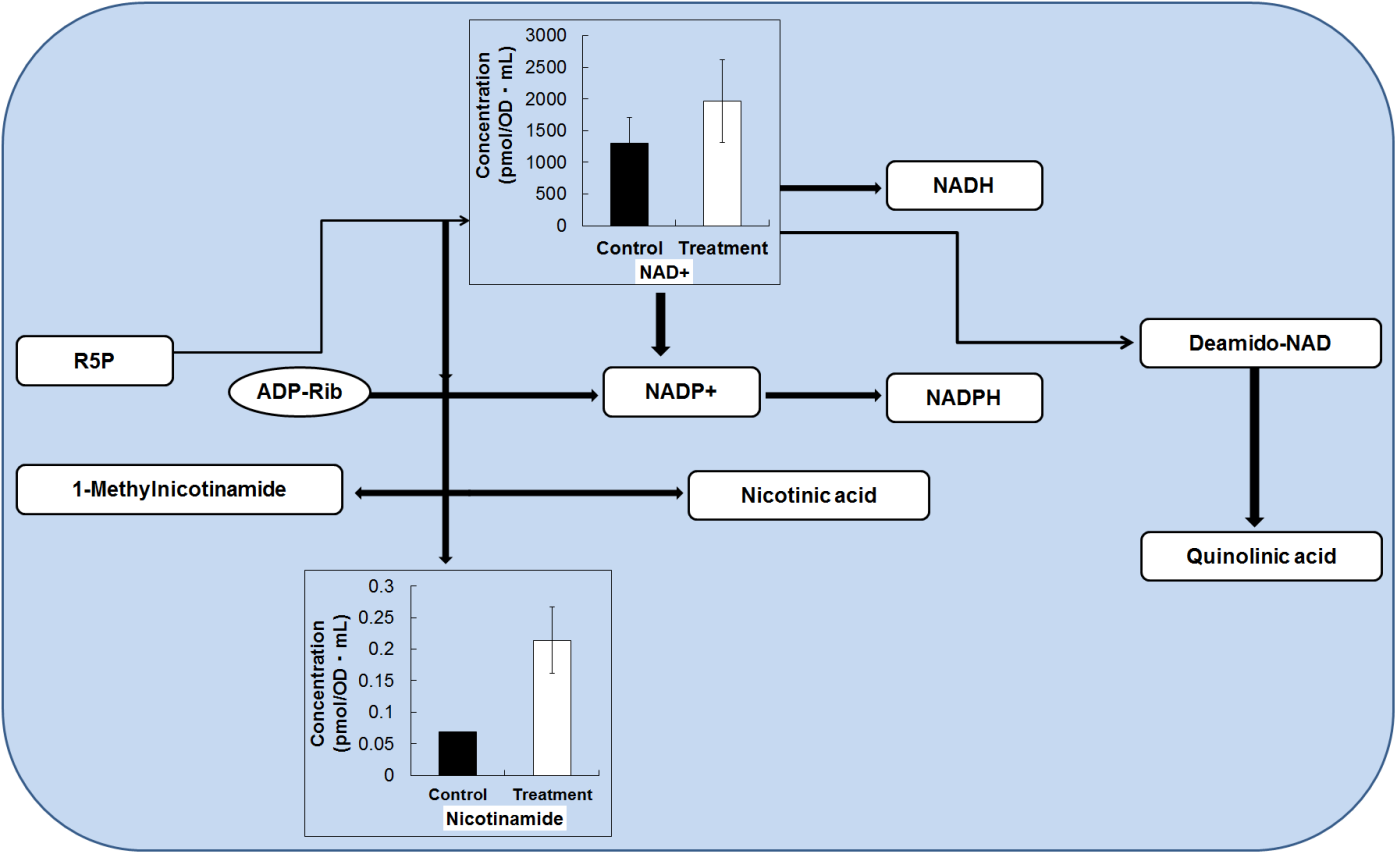
Metabolite changes in the nicotinamide metabolism by *L*. *plantarum* fermentation. Changes in the metabolite levels in the fermented *O*. *japonicus* by *L*. *plantarum* (gray bars) compared with those in the *O*. *japonicus* with *L*. *plantarum* (black bars) determined by capillary electrophoresis time of flight mass spectrometry (CE-TOF-MS). The error bar indicates the mean standard deviation of triplicate samples. The figure also includes a schematic diagram of nicotinamide metabolism.

In our study, the production of the NAD^+^ metabolite was increased by 1.51-fold in the treatment (fermented *O*. *japonicus* by *L*. *plantarum*) compared with the control (*O*. *japonicus* just added *L*. *plantarum*) samples (Fig 4). Thus, the growth of *L*. *plantarum* in presence *O*. *japonicus* could be advantageous because increased NAD^+^ metabolism can boost human health. A growing body of evidence supports the view that NAD^+^ metabolism regulates significant biological processes, as well as having effects on lifespan [32, 33]. The function of NAD^+^ metabolism in fitness and disease has received increased attention as the use of nicotinic acid has emerged as a remedy to manage hyperlipidemias [33]. In addition, nicotinamide can protect tissues. NAD^+^ exerts strong effects throughout the poly (ADP-ribose) polymerases, mono-ADP ribosyltransferases, and the recently discovered sirtuin enzymes. These enzymes regulate metabolism, DNA repair, apoptosis, stress resistance, and endocrine signaling, signifying that NAD^+^ metabolism could be targeted for a wide range of therapies [32; 33]. Production of NAD^+^ is achieved through both recycling and de novo pathways in the majority of microorganisms and in human cells [34]. Nicotinamide and nicotinic acid are clinically useful as pharmacological agents [35]. Currently, nicotinamide is used to prevent type 1 diabetes [36], and neurotoxicity and ischemia [37].

Nicotinamide was first used in 1937 as “pellagra-preventive” agent. It has also contributed to remedial approaches as an “AIDS preventive” agent [38, 39] and a “tuberculosis preventive agent” [39]. The intracellular nicotinamide and NAD^+^ levels have been linked to cellular responses essential for cell survival [40]. Nicotinamide is an active form of Vitamin B3 or niacin [41]. In humans, dietary nicotinamide and niacin are absorbed from stomach and intestine through both sodium-dependent and passive diffusions [42, 43]. A significant deficiency of nicotinamide in humans causes the disease pellagra (Italian *“pelle agra”* means *“*rough skin*”*), which is characterized by photosensitive dermatitis, dementia, diarrhea, and death [44]. The incidence of pellagra has been linked to the lack of NAD^+^ and NADP^+^ levels in maintaining energy for cellular functions [43]. In the CE-TOF-MS analysis, the production of nicotinamide was increased by 3.11-fold in *L*. *plantarum* fermented with *O*. *japonicus* (treatment) compared with the control (Fig 4). In terms of nicotinamide levels, *L*. *plantarum* with *O*. *japonicus* can enhance human health.

Succinic acid is formed in three ways: the tricarboxylic acid (TCA) cycle, which is mostly active in completely anaerobic environments, the oxidative branch of the TCA cycle, which is initially active in aerobic environments, and the glyoxylate shunt, which is active in aerobic environment leading to adaptation to growth on acetate. The aerobic routes synthesize 1 mole of succinic acid from 1 mole of glucose, while the anaerobic pathway outputs 2 moles of succinic acid in combination with fixation of CO_2_. Thus, succinic acid is produced efficiently under anaerobic conditions using the reductive branch of the TCA cycle [45, 46].

**Fig 5:**
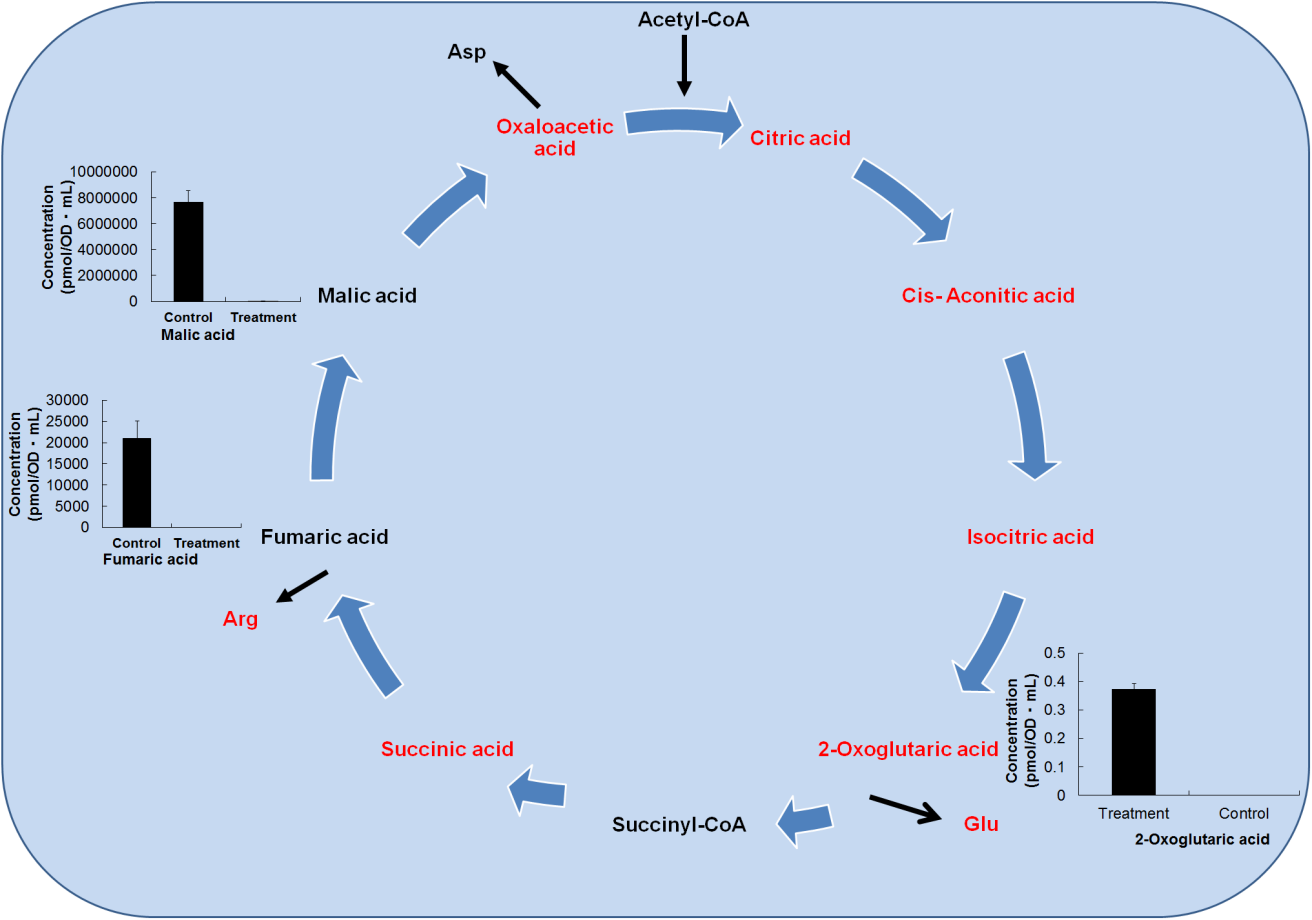
Alteration of the metabolites in the TCA cycle by fermentation. The metabolite levels in TCA cycle in in the fermented *O*. *japonicus* by *L*. *plantarum* (gray bars) compared with those in the *O*. *japonicus* with *L*. *plantarum* (black bars) obtained by capillary electrophoresis time of flight mass spectrometry (CE-TOF-MS). The error bar indicates the mean standard deviation of triplicate samples. The figure also includes a schematic diagram of the TCA cycle.

For the aerobic process, the TCA cycle is a central metabolic pathway that accounts for the most important portion of amino acid, fatty acid, and carbohydrate oxidation to facilitate energy production [47]. The growth of *L*. *plantarum* in the presence *O*. *japonicus* changed the intermediate metabolites of TCA cycle dramatically (Fig 5). In the treatment group, the content of the TCA cycle metabolites, fumaric acid, malic acid, and isocitric acid, was completely reduced compared with their levels in the control. Lactic acid is mainly produced from the conversion of malic acid; therefore, in the treatment group, malic acid is completely converted to lactic acid. By contrast, the level of succinic acid was nearly equal in the control and treatment samples.

The Asp content was 56.16%, the Arg content was 53.20%, and the Glu content was 64.04% in treatment group. Only the 2-oxoglutaric acid (2-OG) content was significantly increased in the treatment group compared with the control. The majority of NADH and FADH2 is produced in the TCA cycle and provides high-energy electrons to the electron transport system for the production of ATP. Thus, variation of metabolites of the TCA cycle would be likely to hamper the production of essential energy molecules. The existence of 2-OG in the diet improved connective tissue in pigs throughout suckling and induced healing of bone and cartilage damage due to prenatal dexamethasone action [48].

**Fig 6:**
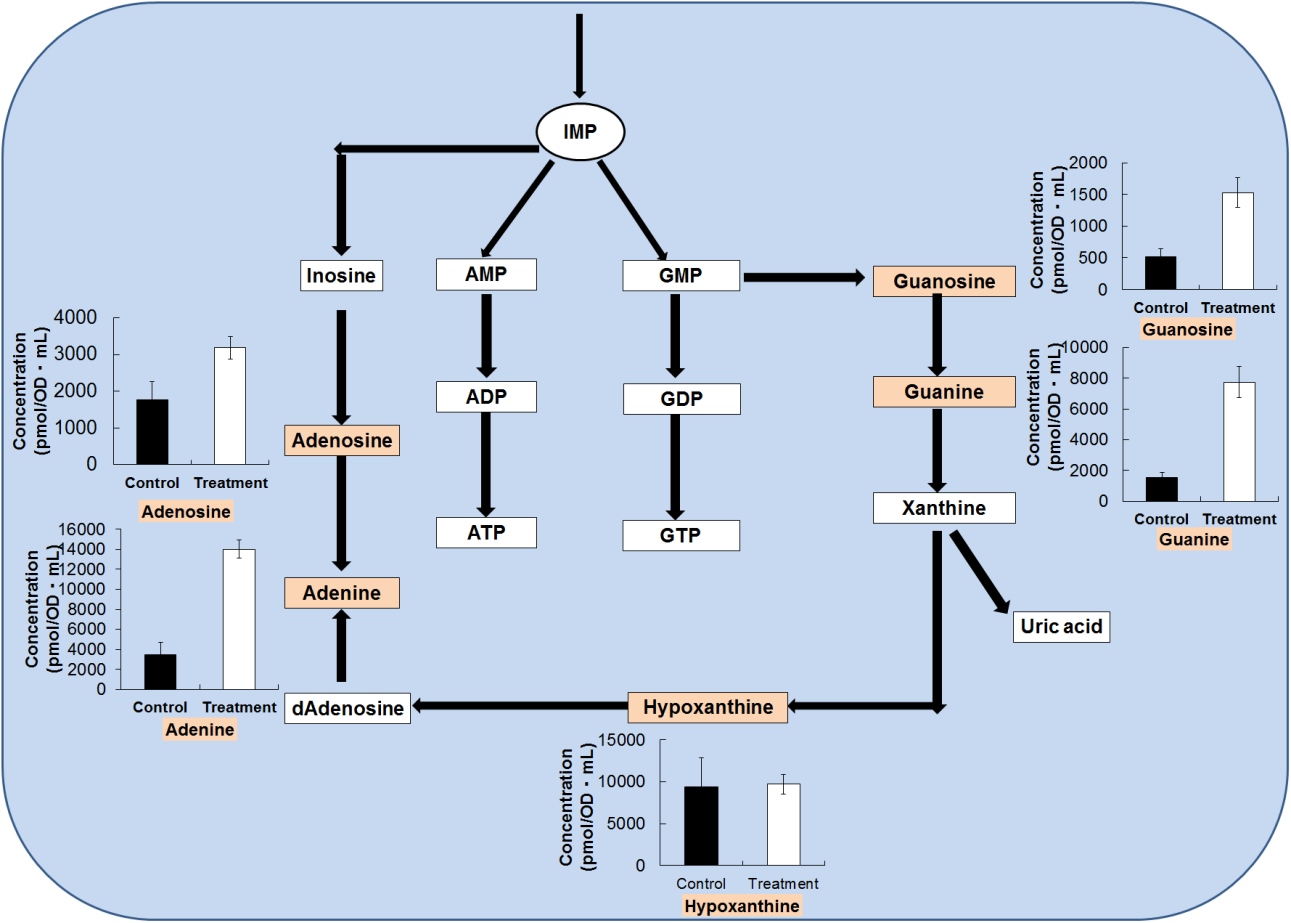
Alteration of the metabolites in the purine nucleotide biosynthesis pathway. The changes in metabolite levels in the fermented *O*. *japonicus* by *L*. *plantarum* (gray bars) compared with those in the *O*. *japonicus* with *L*. *plantarum* (black bars) were elucidated by capillary electrophoresis time of flight mass spectrometry (CE-TOF-MS). The error bars indicate the mean standard deviation of triplicate samples. The figure also includes a schematic diagram of the purine nucleotide biosynthesis pathway.

The growth of *L*. *plantarum* in the presence of *O*. *japonicus* extract caused variation in diverse metabolites of purine biosynthesis (Fig 6). Most of the metabolites of these pathways showed significant increases after fermentation, such as the 1.80- and 2.95-fold increase in the adenosine and guanosine content, respectively. The amounts of adenine and guanine also increased by 4.00- and 5.04-fold, respectively, after fermentation. In case of the pyrimidine metabolites, the level of uracil increased by 4.73-fold after fermentation. Uracil and inosine are essential for the growth of different *Lactobacillus* species [49]. By contrast, the amounts of ADP and ATP were below the detection limit in both the treatment and control groups. Purine and pyrimidine nucleotides are the leading energy carriers and subunits of nucleic acids, and are precursors for the synthesis of nucleotide cofactors such as NAD. The results indicated that ATP was completely exhausted in *O*. *japonicus* fermented with *L*. *plantarum*.

In the fermented *O*. *japonicus*, the content of histamine in the urea cycle-related metabolism was increased significantly by 1.49-fold compared with the control. Histamine is involved in the regulation of several physiological functions, such as cell proliferation, differentiation, regeneration, hematopoiesis, wound healing, and embryonic development. It influences various immune/inflammatory functions [50] and contributes to the development of allergic inflammatory responses by enhancing the secretion of pro-inflammatory cytokines, such as IL-1a, IL-1b, and IL-6, in addition to chemokines like IL-8, in various cell types and tissues [51].

## Conclusions

*L*. *plantarum* is an extremely versatile bacterium that can acclimatize to diverse ecological niches it can ferment diverse types of carbohydrates and sugars. In particular, it can grow in the harsh circumstances of the gastrointestinal tract. Thus, using it as a probiotic can be beneficial to human health. The metabolomics analysis of *O*. *japonicus* fermented by *L*. *plantarum* revealed significant alterations of various metabolites. In the glycolysis pathway, all metabolite levels were reduced except for glycerol-3-phosphate. In the TCA cycle, the levels of all metabolites decreased cycle except for 2-oxoglutaric acid. In the pentose phosphate pathway, there were significant increases in the amount of NAD^+^ and nicotinamide, which are beneficial to human health. Nucleotide metabolites, such as uracil, adenine, adenosine, guanosine, and guanine, also increased. Thus, the metabolome analysis showed that the fermentation of *O*. *japonicus* by *L*. *plantarum* altered many metabolites, some of which can exert beneficial effects on human health.

## Supporting information

S1 Table Intracellular metabolites of glycolysis, pentose phosphate, TCA cycle, and nucleotide metabolism detected from *L*. *plantarum* and *L*. *plantarum* in the presence of *O*. *japonicus* plant extract using capillary electrophoresis time of flight mass spectrometer (CE-TOF-MS) analysis. (DOCX)
