# Supplementary material for: Analysis of metabolomic profile of fermented Orostachys japonicus A. Berger by capillary electrophoresis time of flight mass spectrometry
Source: PLoS One. 2017 Jul 13;12(7):e0181280. doi: 10.1371/journal.pone.0181280 (PMC5509444; doi:10.1371/journal.pone.0181280)
Supplement: S1 Table — (DOCX) [file pone.0181280.s001.docx]

**Supporting information**

**S1 Table. Intracellular metabolites of glycolysis, pentose phosphate, TCA cycle, and nucleotide metabolism detected from *L. plantarum* and *L. plantarum* in the presence of *O. japonicus* plant extract using capillary electrophoresis time of flight mass spectrometer (CE-TOF-MS) analysis**

| **Comparative Analysis** | | | | | | | | |  |
| --- | --- | --- | --- | --- | --- | --- | --- | --- | --- |
|  |  |  |  | **Control** |  | **Treatment** |  | **Treatment vs. Control** |  |
| **ID** | **HMT DB † Compound name** | **m/z** | **MT** | **Mean** | **S.D.** | **Mean** | **S.D.** | **Ratio ^¶^** | ***p*-value ^\|\|^** |
| A_0056 | 2-Phosphoglyceric acid | 184.986 | 19.09 | 0.109709 | 0.026687 | N.D. | N.A. | <1 | N.A. |
| A_0048 | *cis*-Aconitic acid | 173.009 | 26.01 | 7.17736 | 1.184629 | 0.544022 | 0.07961 | 0.08 | 0.010* |
| A_0064 | Citric acid | 191.02 | 24.25 | 142.565 | 19.673049 | 26.301437 | 1.649746 | 0.2 | 0.009** |
| A_0093 | Fructose 1,6-diphosphate | 338.989 | 14.81 | 0.110488 | 0.052594 | 0.038718 | 0.003556 | 0.4 | 0.141 |
| A_0086 | *N*-Acetylglucosamine 1-phosphate | 300.048 | 9.74 | 0.1008 | 0.035499 | 0.065714 | 0.005795 | 0.7 | 0.227 |
| C_0111 | Glucosamine | 180.086 | 9.68 | 0.335648 | 0.155934 | 0.298319 | 0.065614 | 0.9 | 0.731 |
| A_0066 | Glucuronic acid | 193.034 | 8.28 | 0.391702 | N.A. | N.D. | N.A. | <1 | N.A. |
| A_0063 | Isocitric acid | 191.02 | 25.78 | 523.943 | 55.5347 | 12.7341 | 1.57682 | 0.02 | 0.004** |
| C_0129 | *N*-Acetylmannosamine | 222.098 | 22.95 | 1.173546 | 0.243606 | 0.252097 | 0.044083 | 0.2 | 0.020* |
| A_0115 | UDP-glucose | 565.051 | 8.86 | 0.105464 | 0.012031 | N.D. | N.A. | <1 | N.A. |
| A_0116 | UDP-*N*-acetylglucosamine | 606.079 | 8.65 | 0.043872 | 0.013927 | N.D. | N.A. | <1 | N.A. |
| C_0090 | 2-Aminoadipic acid | 162.076 | 11.36 | 0.30363 | 0.069945 | 0.06882 | 0.024775 | 0.2 | 0.018* |
| A_0007 | 2-Hydroxybutyric acid | 103.041 | 9.89 | 0.557834 | 0.076685 | 0.287501 | 0.021597 | 0.5 | 0.020* |
| C_0054 | 5-Amino-4-oxovaleric acid | 132.065 | 8.35 | 32.83752 | 4.885204 | 0.402625 | 0.090853 | 0.012 | 0.007** |
| C_0093 | 5-Hydroxylysine | 163.107 | 7.48 | 0.090433 | 0.038053 | 0.08368 | 0.011497 | 0.9 | 0.792 |
| C_0074 | Acetylcholine | 146.117 | 7.94 | 9.309541 | 4.114804 | 9.707249 | 1.76873 | 1 | 0.888 |
| C_0037 | Betaine | 118.086 | 11.76 | 22.107983 | 9.401314 | 13.975403 | 2.287115 | 0.6 | 0.27 |
| C_0092 | Carnitine | 162.111 | 8.9 | 0.492546 | 0.215954 | 0.319168 | 0.057777 | 0.6 | 0.297 |
| C_0025 | Choline | 104.107 | 7.16 | 23.60655 | 4.782771 | 1.283108 | 0.189509 | 0.05 | 0.015* |
| A_0002 | Glycolic acid | 75.01 | 12.93 | 0.638896 | 0.179889 | 0.383168 | 0.040144 | 0.6 | 0.127 |
| C_0139 | Glycerophosphocholine | 258.11 | 22.51 | 2.068642 | 1.007345 | 0.304208 | 0.050766 | 0.15 | 0.093 |
| C_0077 | Lys | 147.112 | 7.19 | 22.446549 | 10.383067 | 18.249194 | 2.478511 | 0.8 | 0.56 |
| C_0082 | Met | 150.058 | 11.19 | 6.499831 | 3.289529 | 5.528923 | 1.138353 | 0.9 | 0.669 |
| C_0113 | Phosphorylcholine | 184.073 | 21.41 | 0.657811 | 0.211013 | 0.153561 | 0.033219 | 0.2 | 0.051 |
| C_0048 | Pipecolic acid | 130.086 | 10.72 | 0.672437 | 0.224686 | 0.318682 | 0.056569 | 0.5 | 0.104 |
| C_0145 | Saccharopine | 277.139 | 11.2 | 0.522259 | 0.156495 | 0.30004 | 0.034163 | 0.6 | 0.127 |
| C_0154 | *S*-Adenosylmethionine | 399.143 | 7.43 | 0.026933 | 0.006668 | 0.028219 | 0.005751 | 1 | 0.813 |
| C_0026 | Ser | 106.05 | 10.41 | 11.785555 | 4.798383 | 8.310481 | 1.855423 | 0.7 | 0.339 |
| C_0038 | Thr | 120.065 | 10.97 | 12.480754 | 5.112177 | 9.539045 | 1.805407 | 0.8 | 0.429 |
| C_0118 | *N*^6^,*N*^6^,*N*^6^-Trimethyllysine | 189.159 | 7.51 | 0.358308 | 0.089012 | 0.085519 | 0.019405 | 0.2 | 0.029* |
| A_0008 | Glyceric acid | 105.019 | 11.03 | 0.315435 | 0.056106 | 0.12598 | 0.011667 | 0.4 | 0.024065* |
| C_0064 | 1-Methylnicotinamide | 137.07 | 7.7 | 0.049632 | 0.009383 | 0.045585 | 0.00736 | 0.9 | 0.663 |
| A_0092 | Ascorbate 2-glucoside | 337.079 | 7.29 | 0.478093 | 0.051042 | 0.017809 | N.A. | 0.04 | N.A. |
| A_0053 | Ascorbic acid | 175.025 | 8.56 | 1.260868 | 0.1933416 | N.D. | N.A. | <1 | N.A. |
| C_0043 | Nicotinamide | 123.056 | 7.72 | 0.068756 | 0.022643 | 0.213981 | 0.052862 | 3.1 | 0.027* |
| C_0044 | Nicotinic acid | 124.039 | 10.42 | 1.28346 | 0.264259 | 1.1064689 | 0.179095 | 0.9 | 0.398 |
| A_0075 | Pantothenic acid | 218.105 | 7.7 | 0.241183 | 0.034135 | 0.089081 | 0.004254 | 0.4 | 0.015* |
| C_0098 | Pyridoxal | 168.066 | 9.05 | 0.038247 | 0.002739 | 0.02879 | 0.005202 | 0.8 | 0.068 |
| C_0101 | Pyridoxine | 170.08 | 9.04 | 0.113958 | 0.02795 | 0.050602 | 0.011948 | 0.4 | 0.043* |
| C_0061 | Adenine | 136.062 | 7.96 | 1.092687 | 0.368557 | 4.381607 | 0.283258 | 4 | 0.00036626*** |
| C_0142 | Adenosine | 268.103 | 10.31 | 0.575231 | 0.165789 | 1.039114 | 0.102296 | 1.8 | 0.021* |
| A_0095 | AMP | 346.057 | 9.75 | 0.1333 | 0.044799 | N.D. | N.A. | <1 | N.A. |
| A_0091 | cAMP | 328.045 | 7.63 | 0.165558 | 0.066079 | 0.050537 | 0.008907 | 0.3 | 0.092 |
| A_0094 | cGMP | 344.042 | 7.83 | 0.5215102 | 0.218708 | 0.116275 | 0.011742 | 0.2 | 0.084 |
| A_0089 | CMP | 322.047 | 9.82 | 0.168444 | 0.079143 | 0.09063 | 0.007089 | 0.5 | 0.23 |
| C_0136 | 2'-Deoxyadenosine | 252.11 | 10.07 | 2.70E-02 | 1.20E-02 | 9.90E-02 | 1.30E-02 | 3.6 | 0.002** |
| C_0141 | 2'-Deoxyguanosine | 268.103 | 12.01 | 0.033302 | N.A. | 0.0220922 | 0.0057823 | 0.7 | N.A. |
| A_0094 | cGMP | 344.042 | 7.83 | 0.52151 | 0.218708 | 0.116275 | 0.011742 | 0.2 | 0.084 |
| C_0085 | Guanine | 152.058 | 8.67 | 3.50E-01 | 8.20E-02 | 1.80E+00 | 2.30E-01 | 5 | 0.004** |
| C_0147 | Guanosine | 284.098 | 13 | 1.20E-01 | 2.90E-02 | 3.50E-01 | 5.40E-02 | 3 | 0.007** |
| C_0104 | 3-Methylhistidine | 170.092 | 7.82 | 0.058549 | 0.008415 | 0.028577 | 0.005381 | 0.5 | 0.072 |
| C_0108 | Citrulline | 176.102 | 11.53 | 1.025407 | 0.376798 | 0.486769 | 0.106052 | 0.5 | 0.123 |
| C_0022 | GABA | 104.071 | 8.03 | 14.114368 | 5.299382 | 8.637284 | 1.377872 | 0.6 | 0.21 |
| C_0076 | Gln | 147.076 | 11.22 | 37.576486 | 6.872089 | 0.545083 | 0.132523 | 0.015 | 0.011* |
| C_0151 | Glutathione (GSH) | 308.09 | 13.79 | 0.524606 | 0.051925 | N.D. | N.A. | <1 | N.A. |
| C_0150 | Glutathione (GSSG)_divalent | 307.082 | 12.69 | 0.062075 | 0.021876 | N.D. | N.A. | <1 | N.A. |
| C_0086 | His | 156.076 | 7.62 | 4.884853 | 1.548666 | 2.485647 | 0.330118 | 0.5 | 0.11 |
| C_0031 | Histamine | 112.087 | 4.97 | 0.053646 | 0.023255 | 0.079892 | 0.013222 | 1.5 | 0.183 |
| C_0053 | Hydroxyproline | 132.065 | 12.54 | 0.191864 | 0.072002 | 0.081709 | 0.013673 | 0.4 | 0.113 |
| C_0148 | 5'-Deoxy-5'-methylthioadenosine | 298.095 | 10.51 | 0.031383 | N.A. | 0.017937 | 0.002727 | 0.6 | N.A. |
| A_0052 | *N*-Acetylaspartic acid | 174.042 | 14.81 | 0.07429 | 0.022571 | 0.039432 | 0.0040732 | 0.5 | 0.112 |
| C_0059 | Ornithine | 133.097 | 7.12 | 2.288381 | 1.158051 | 1.720966 | 0.228666 | 0.8 | 0.487 |
| A_0019 | 5-Oxoproline | 128.036 | 9.77 | 9.9010206 | 3.522632 | 5.924515 | 0.567839 | 0.6 | 0.187 |
| C_0035 | Pro | 116.07 | 11.28 | 15.716911 | 6.726532 | 13.341544 | 2.497659 | 0.8 | 0.613 |
| C_0011 | Putrescine | 89.107 | 4.9 | 0.686709 | 0.097658 | 0.101312 | 0.003212 | 0.15 | 0.009** |
| C_0075 | Spermidine | 146.165 | 4.71 | 0.53789 | 0.148491 | 0.013763 | 0.002686 | 0.03 | 0.026* |
| C_0124 | Spermine | 203.22 | 4.65 | 0.049727 | 0.016696 | N.D. | N.A. | <1 | N.A. |
| C_0001 | Urea | 61.041 | 21.96 | 1.476662 | 0.513616 | 0.851067 | 0.252783 | 0.6 | 0.157 |

ID consists of analysis mode and number. 'C' and 'A' showed cation and anion modes, respectively

N.D. (Not Detected): The metabolite was below the detection limits.

N.A. (Not Available): The calculation was not possible.

^†^ Putative metabolites that were assigned on the basis of *m/z* and migration time (MT); HMT DB, Human Metabolome Technologies database

^¶^ In the ratio calculation, the latter was denominator.

^||^ The p-value in Welch's t-test. * < 0.05, ** < 0.01, *** < 0.001

They were sorted by the ratio of treatment to control in descending order.
